# Opsins are phospholipid scramblases in all domains of life

**DOI:** 10.1128/mbio.03278-25

**Published:** 2025-11-26

**Authors:** Zachary A. Maschmann, David E. Hardy, Indu Menon, Jenna Webb, Anant K. Menon, Katrina T. Forest

**Affiliations:** 1 Department of Bacteriology, University of Wisconsin-Madison 205263, Madison, Wisconsin, USA; 2 Department of Biochemistry, Weill Cornell Medical College 12295 , New York, New York, USA; Massachusetts Institute of Technology, Cambridge, Massachusetts, USA

**Keywords:** actinorhodopsin, ActR, bacteriorhodopsin, CGMD, lipid scrambling, membrane biogenesis, photoheterotrophy, rhodopsin

## Abstract

Opsins are highly abundant retinal proteins in the membranes of photoheterotrophic bacteria. However, some microbial genomes encode an *opsin* but lack the gene for the final enzyme in retinal synthesis. To account for this paradox, we hypothesized that bacterial opsins play a role in membrane structure and/or biogenesis independent of their potential for light-driven signaling or proton pumping. After purifying actinorhodopsin from a cell-free expression system and from *Escherichia coli* membranes upon overexpression, we demonstrated both *in vitro* and *in silico* that actinorhodopsin from *Nanopelagicus ca*. is a phospholipid scramblase, serving in its pentameric state as a retinal-independent phospholipid diffusion channel. Phospholipid headgroups move along a transbilayer path between actinorhodopsin protomers to equilibrate lipid content in the inner and outer leaflets. Two profound activities, membrane biosynthesis and capture of light energy, are thus facilitated by one ancient bacterial polypeptide. Light-dependent activity and light-independent phospholipid scrambling are shared functions of eukaryotic, archaeal, and bacterial rhodopsins.

**IMPORTANCE:**

Cells are surrounded by membranes that concentrate metabolites and protect cellular contents. Most biomembranes are phospholipid bilayers, in which the phospholipids of each leaflet orient their greasy tails inward and polar groups outward. Bilayer biogenesis depends on phospholipids synthesized on the cytofacial side of the membrane reorienting to the extracellular membrane leaflet. This reorientation requires proteins, termed scramblases, and it was shown that rhodopsins—7-helix photoactive membrane proteins bound to the cofactor retinal—from organisms as widely divergent as mammals and archaea possess scramblase activity. Now we conclusively demonstrate, using purified proteins in laboratory membranes as well as computational approaches, that bacterial rhodopsins are also phospholipid scramblases. This work is important because it highlights a surprising commonality among bacteria, archaea, and eukaryotes and because it shows that rhodopsins—ancient proteins found in the last universal common ancestor—manifest two seemingly unrelated biochemical functions in one protein.

## INTRODUCTION

Living cells engage in an ongoing process of self-construction. Phospholipid bilayer membranes separate the cellular interior from the exterior, concentrate cytoplasmic contents, and protect cellular components. Membrane production is needed for cell division and as part of turnover and repair processes (1). Phospholipids are synthesized in the cytoplasmic leaflet of biogenic membranes, such as the cytoplasmic membrane of bacteria and the endoplasmic reticulum (2). Reorientation of phospholipids between leaflets, known as *lipid scrambling,* is necessary for the uniform growth of the bilayer but occurs too slowly to support life if unassisted (3–8). Biogenesis of all extant membrane bilayers requires a solution to this problem (9, 10), and the evolution of this function likely would have provided ancient protocells with strong fitness benefits. Indeed, compartmentalization to support early life may have relied on the modularity and renewability of lipid bilayers (11, 12). Living cells use a facilitated lipid diffusion channel, termed a scramblase, to catalyze transbilayer phospholipid equilibration (10, 13–16). Scramblases are thought to shield the charged phospholipid head group from the hydrophobic bilayer interior to expedite lipid scrambling (10, 13–16).

In recent years, rhodopsins—well known for their photon capture-based activities—have been shown to moonlight as scramblases. Menon and colleagues (17) used a fluorescence-based assay to show that purified bovine rhodopsin exhibits scramblase activity when reconstituted into large unilamellar vesicles, accounting for previous observations that lipids scramble rapidly across photoreceptor disc membranes (18, 19). Scramblase activity was shown to be ATP-independent and constitutive, occurring in the absence of light or the retinal chromophore. Subsequent work showed that an archaeal rhodopsin—bacteriorhodopsin (BR) from the archaeon *Halobacterium salinarum*—also had light-independent scramblase activity (20). This functional parallelism was not a foregone conclusion, given that the mammalian visual sensory transducer rhodopsin and archaeal proton-pumping BR are found in anciently diverged organisms that differ greatly in membrane composition, architecture, metabolism, and environment.

Eubacterial rhodopsins have been known only since Sargasso Sea metagenomics efforts uncovered proton-pumping proteorhodopsins in marine bacteria in 2000 (21), followed by the identification of opsin gene sequences in highly abundant nano-actinobacteria in fresh water (22). Like BR, these bacterial retinylidene opsins harness photon energy to pump protons across the membrane (23–25). Yet, notably, not all opsin-encoding bacteria carry genes for the complete enzymatic pathway to synthesize the retinal chromophore (23, 24). While retinal may be scavenged in some natural environments (26–28), the incomplete pathway nonetheless led us to hypothesize that actinobacterial opsins (ActR) may moonlight (29) and exhibit a second, retinal-independent function. Given that *actR* transcript levels in the natural environment are extremely high (23, 30), implying abundant protein levels, this function could be related to membrane structure and stability, echoing the fact that BR makes up 75% of the dry weight of *Halobacterium salinarum* purple membranes (31). Alternatively, given that bovine visual opsin and BR are identified scramblases (17, 20), we hypothesized that ActR and other bacterial opsins would also be phospholipid scramblases. This idea was bolstered by recent preliminary evidence of a gram-negative bacterial proteorhodopsin (ptqPR) from *Psychroflexus torquis* acting as a scramblase (32).

We now show that ActR catalyzes the interchange of phospholipid between bilayer leaflets. The dual ActR functions of proton pump (23) and scramblase emphasize the versatility of photoreceptor function and may provide a molecular connection between photoheterotrophy and membrane biogenesis.

## RESULTS

### Large-scale synthesis of ActR in an *in vitro* transcription/translation system

**Fig 1:**
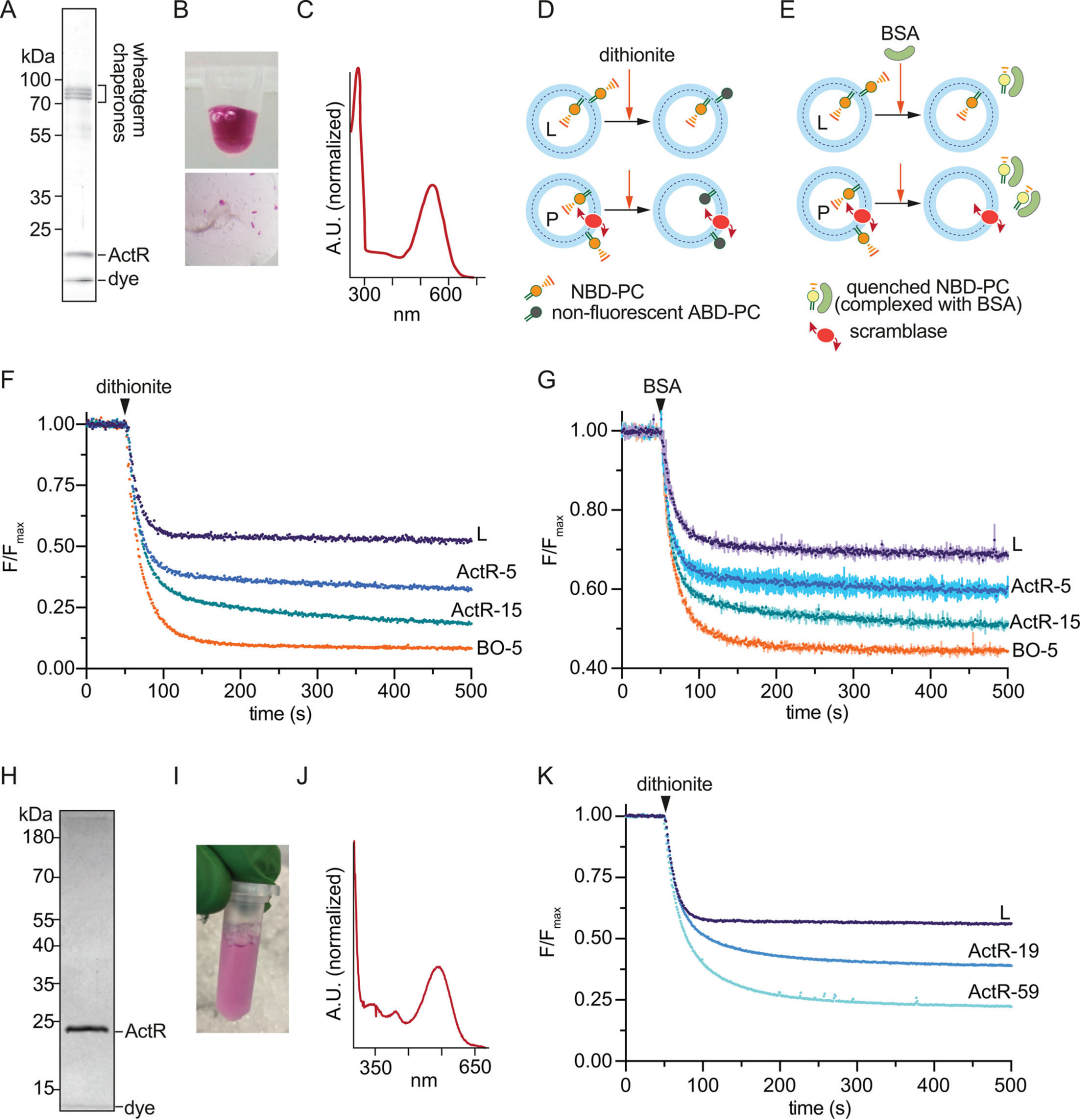
acI-B (*Nanopelagicus ca*.) ActR expressed in a cell-free system or in *Escherichia coli* is a phospholipid scramblase. (**A and H**) SDS-PAGE analysis of affinity-purified (**A**) cell-free- or (**H**) *E. coli*-produced ActR following size-exclusion chromatography. (**B and I**) Image of (**B**) cell-free- or (**I**) *E. coli*-produced ActR showing strong purple color. (**C and J**) Absorbance spectrum of (**C**) cell-free- or (**J**) *E. coli*-produced ActR. (**D**) Protein-free liposomes (L) and scramblase-containing proteoliposomes (P) are shown schematically. The vesicles are prepared with a trace quantity of 4-nitro-2,1,3-benzoxadiazole phosphatidylcholine (NBD-PC) distributed equally between inner and outer leaflets. Upon addition of dithionite, the nitro group in NBD-PC is covalently modified to an amino group, and the resulting ABD-PC is non-fluorescent. Liposomes retain fluorescence signal from NBD-PC in the inner leaflet as dithionite cannot enter the vesicles; for scramblase-containing proteoliposomes, lipids in the inner and outer leaflets exchange and fluorescence decays further as NBD-PC molecules in the inner leaflet reach the outer leaflet where they are modified by dithionite. (**E**) As in (**D**), except that fatty-acid-free BSA is used as a topological probe. NBD-PC molecules that desorb from the outer leaflet are captured by BSA. In complex with BSA, NBD-PC fluorescence is ~60% lower than when the lipid is in the membrane. (**F, G, and K**) Normalized time course scramblase assay data for (**F and G**) cell-free- or (**K**) *E. coli*-produced ActR. Dithionite or BSA was added at the indicated time point. Error bars in panel **G** represent the standard deviation of duplicate measurements; data in panels **F and G** represent one of three independent experiments carried out under different proteoliposome reconstitution conditions, and data in panel **K** represent one of four independent experiments. Proteoliposomes were generated using ActR or bovine opsin (BO) at different protein/phospholipid ratios (units of µg/µmol) as indicated in the trace labels, e.g., ActR-5 and ActR-15 represent samples with protein/phospholipid ratios of 5 and 15, respectively.

We synthesized ActR (*Nanopelagicus ca*. genome sequence L06) in a wheat-germ extract translation system (33) with the inclusion of retinal in the translation reaction. Purification by nickel affinity and size exclusion chromatography yielded a single ActR protein band that migrated more rapidly (<25 kDa) than expected from its ~29 kDa molecular weight and co-purified with a triplet of proteins identified by mass spectrometry as chaperones from the wheat germ extract (Fig. 1A). The protein was highly colored (Fig. 1B) with peak absorbance at 544 nm—characteristic of a bacterial rhodopsin with leucine at the tuning position, and in agreement with our previous observations for bacterially produced ActR (23, 34) (Fig. 1C). Purity and homogeneity of ActR were further demonstrated by our ability to crystallize it using a cubic lipidic phase (Fig. 1B, bottom panel).

### ActR facilitates transbilayer phospholipid transport

To determine the scramblase activity of ActR, we used standard fluorescence-based assays (Fig. 1D and E) in which proteins are reconstituted into large unilamellar vesicles containing a trace amount of a fluorescent phospholipid reporter, e.g., 4-nitro-2,1,3-benzoxadiazole phosphatidylcholine (NBD-PC) (17, 20, 35). To assay scrambling, the vesicles are interrogated with topological probes—the dianion dithionite (Fig. 1D), which reacts with the NBD fluorophore to eliminate its fluorescence, and fatty acid-free BSA (Fig. 1E), which captures NBD-PC molecules that desorb from the vesicle surface, resulting in partial fluorescence quenching.

Treatment of protein-free vesicles with dithionite is expected to result in ~50% fluorescence loss as NBD-PC molecules in the outer leaflet of the vesicles are modified, whereas those in the inner leaflet are protected (Fig. 1D, top row). This is what is experimentally observed (Fig. 1F, trace labeled “L” plateaus at 53%). However, when the vesicles contain an active scramblase, rapid exchange of NBD-PC between the inner and outer leaflets of the vesicle results in total loss of fluorescence as the entire complement of NBD-PC becomes exposed to dithionite (Fig. 1D, bottom row). We reconstituted two different amounts of cell-free produced ActR via detergent-destabilization of preformed liposomes. We also reconstituted bovine opsin (BO) as a positive control. As shown in Fig. 1F, dithionite treatment of ActR proteoliposomes (ActR-5 and ActR-15) showed a greater loss of fluorescence than seen with empty vesicles (L), indicating that ActR has scramblase activity. The addition of hydroxylamine, which disrupts the Schiff’s base linkage to retinal, had no impact on scrambling activity (data not shown).

The reconstitutions were done at a protein:phospholipid ratio of ~5 µg/µmol (ActR-5 and BO-5) or ~15 µg/µmol (ActR-15). As ActR is smaller than BO (~29 vs ~40 kDa), it would be expected to functionalize at least as many vesicles as BO when reconstituted at ~5 µg/µmol. Yet, the data show that the extent of fluorescence reduction is not as great for ActR-5 (73%) as for BO-5 (91%) when the proteins are similarly reconstituted (Fig. 1F). Even when 3× more ActR was reconstituted (ActR-15, Fig. 1F), the extent of reduction was ~84%. A possible explanation for the lower efficiency with which ActR reconstitutes is that, like other opsins (20, 36–38), ActR multimerizes, and protein multimerization *en route* to reconstitution leads to a fraction of vesicles populated with trimers or monomers rather than with functional pentameric ActR scramblases as discussed below.

The fluorescence decay traces for ActR and BO-containing vesicles are well fit with a double-exponential decay function, which represents a convolution of the dithionite reduction reaction rate (*t*_1/2_ ~ 12 s) and a slower scrambling rate. For BO-5, the slow phase has *t*_1/2_ ~ 40 s, whereas for ActR-5 and ActR-15, the *t*_1/2_ values are ~400 and ~180 s, respectively. These values suggest that ActR-mediated scrambling is somewhat slower under these experimental conditions than that observed for BO.

To complement data generated using dithionite, we next used BSA as a topological probe. For empty vesicles, ~30% fluorescence loss is expected, as NBD-PC from the outer leaflet of the vesicles is quantitatively captured by BSA (Fig. 1E, top row). This is because when bound to BSA, the quantum efficiency of NBD-PC is ~60% lower than when the lipid is in the membrane (8, 39). For scramblase-equipped vesicles, transbilayer exchange of NBD-PC enables all NBD-PC molecules to be captured by BSA, resulting in ~60% expected fluorescence loss. Figure 1G shows greater fluorescence loss in ActR-containing vesicles compared with empty vesicles, consistent with their scramblase activity. As with the dithionite assay, greater fluorescence reduction was seen with ActR-15 than with ActR-5, and BO-5 vesicles showed close to the maximum expected fluorescence loss.

### Scramblase activity of ActR purified from *E. coli*

As an orthogonal means to isolate ActR and avoid the co-purification of wheat germ chaperones, we overexpressed the same ActR sequence in *E. coli*, induced expression, added retinal concomitantly, and purified the protein using metal affinity and gel filtration chromatographies. SDS-PAGE analysis revealed a single band running at the expected size for an ActR monomer (Fig. 1H), with a characteristic purple color (Fig. 1I) and a maximum absorbance at 544 nm (Fig. 1J). On reconstitution into unilamellar vesicles, the bacterially produced protein also showed robust phospholipid scramblase activity, measured using the dithionite assay (Fig. 1D and K). Thus, whether produced in a cell-free system or expressed in *E. coli*, ActR demonstrates scramblase activity.

### ActR oligomeric state observed by EM

**Fig 2:**
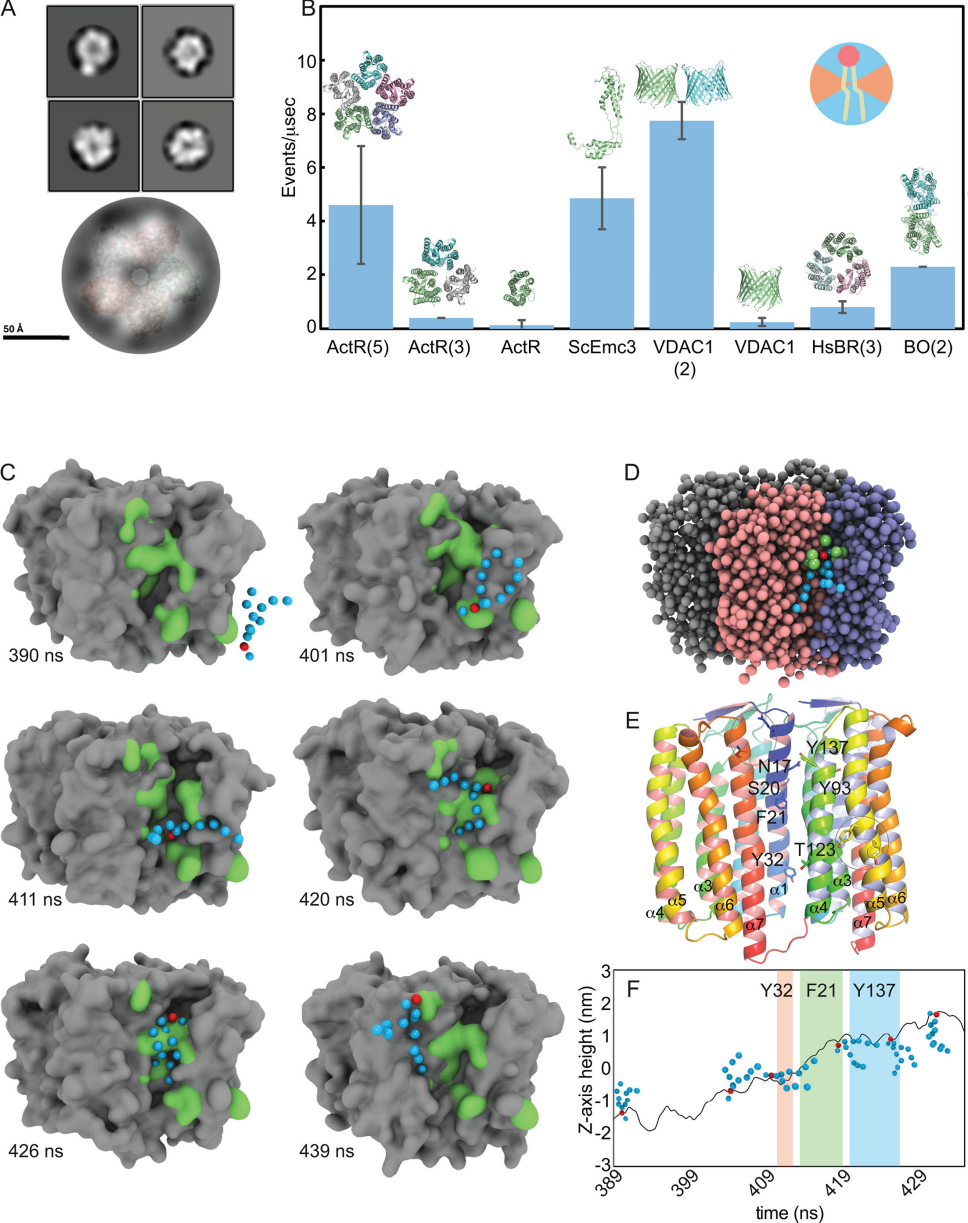
ActR is a pentamer that facilitates scrambling as assessed computationally. (**A**) Array of four representative negative stain electron microscopy images of ActR at pH 7.4, and superposition of representative ActR TEM image over predicted ActR pentameric structure. (**B**) Coarse-grained molecular dynamics (CGMD) scramblase events/µsecond accompanied by structural images. The upper right inset shows the angles (in orange) in which rotations were not counted as scrambling events. (**C**) CGMD scramblase “flip book” images of one dioleoylphosphatidylcholine (DOPC) moving through cleft and re-orienting lipid tails. Those beads that were within 5 Å of the phosphate bead in any frame are shown in green, PO4 bead is red, and lipid tail beads are blue. (**D**) One snapshot from the trajectory represented in part C is shown with beads from each opsin monomer shaded differently to highlight the inter-protomer location of the groove. Green protein beads are all those within 5 Å of the red phosphate bead (in this frame, representing atoms from N17, S20, F21, and Y93), while blue beads depict the lipid tail. (**E**) Ribbon diagram of two adjacent protomers that form the scrambling groove, with side chains in the groove that interact with lipids during scrambling labeled, and occasionally observed interactions circled but not labeled. Each monomer is colored in a reverse rainbow from blue to red as the chain progresses from N to C terminus. Additionally, the left chain has a salmon interior surface, while the right chain has a light blue interior surface, with visible α helices labeled on both chains. (**F**) *Z*-axis height above or below the center of the membrane for the PO4 bead as a function of time during passage through the cleft of the example individual DOPC shown in panel **C**. Dwell times for Y32, F21, and Y137 are depicted as colored bars.

Bacterial opsins have been observed in monomeric, trimeric, and pentameric quaternary arrangements (37, 38, 40) as well as hexamers, although the latter may be due to native signal peptide sequences included in heterologously expressed proteins (26, 41). Extended helices and a 3-omega motif dictate the pentameric state of *Gloeobacter* rhodopsin (38). As these secondary structures are also found in ActR, we expect it to be pentameric. The multimer state of ActR may be important for both its light-dependent proton-pumping and scramblase activities. We investigated the ActR oligomeric state at the neutral pH under which the scramblase assays were carried out, using negative stain transmission electron microscopy (TEM) and analytical size-exclusion chromatography. TEM images indicate a heterogeneous mixture of ActR oligomers (Fig. S2) with pentamers identified in preliminary single particle analysis (Fig. 2A). Pentamers and trimers were both detected by size exclusion chromatography (Fig. S2), likely in equilibrium based on experimental conditions. Moreover, we observed a shift from pentamer and trimer to trimer and monomer at low pH (see further discussion in Supplemental Material). Given experimental observations and the knowledge that native functional states and the most common structural assemblages of proton-pumping and ion-pumping bacterial rhodopsins are pentamers (26, 37, 38, 40, 42), we hypothesize that the pentameric state of ActR is the scramblase active one.

### Coarse-grained molecular dynamics support ActR pentamer scramblase activity

To support our laboratory-based observations that ActR is a lipid scramblase, we employed coarse-grained molecular dynamics (CGMD) simulations over an extended time window to assess the scrambling activity of ActR in three oligomeric states. We used the computational protocol of Li et al. (43) to determine the scrambling rate by counting the number of phospholipid inversions in the membrane per unit time. Three replicates of an ActR pentamer model in a dioleoylphosphatidylcholine (DOPC) membrane bilayer showed ~4 scrambling events/µs (Fig. 2B), consistent with rates calculated for archaeal BR (20). The lipid inversion rates of two positive controls (scEMC3 with ~5 events/µs and VDAC1 dimer with ~8 events/µs) were consistent with the results reported by Li et al. (43). In contrast, the ActR monomer, ActR trimer, and VDAC1 monomer displayed negligible scrambling activity (Fig. 2B).

Lipid translocation by the ActR pentamer shows no directional bias, and although durations vary, on average, the complete inversion takes on the order of 100 ns. Our model suggests that transbilayer phospholipid diffusion occurs at protomer interfaces in ActR and involves polar and aromatic residues that form a hydrophilic groove, consistent with the previously described credit card model (15). Side chains spanning the hydrophilic groove derive from α-helix 1 (α1) of one protomer and α3 and α4 of the adjacent protomer (Fig. 2C through E).

The scrambling trajectory can be described in steps. First, lipids are recruited by polar residues near the bilayer surface. For example, Tyr161 and Tyr164 on α5 serve this role in the event depicted in Fig. 2C. The phospholipid does not linger in these positions for more than a frame or two during the CGMD simulation, and from 20 scrambling events chosen for a deeper analysis (of a total of 138 observed), these residues come into play in only one or two of the events, respectively. Thus, the recruitment step is transient, and many different side chains may be able to act at this step.

After this initial recruitment of the phospholipid, the heart of the scrambling mechanism takes place within the inter-subunit groove. The phosphate bead enters a lower vestibule and contacts Tyr32 (75% of events) and Thr123 (95% of events) (Fig. 2C and E). During this step, the lipid tails may sample an equatorial orientation relative to the bulk lipid of the membrane (Fig. 2C, 411 ns and 420 ns panels). Subsequently, Phe21 (100% of 20 events) passes the DOPC molecule from the lower to the upper pocket. Passage into and through this second water-containing cavity formed by Asn17, Ser20, Tyr93, and Tyr137 (contacted in 90%, 95%, 40%, and 85% of events, respectively) is slow, with variable contact times on the order of 10 ns for these residues (Fig. 2C and F). Water molecules are displaced from or shift within this major pocket when the PO4 bead enters. All of these transitions can include backsliding. The upper cavity is formed by side chains from three helices at the oligomer interface: Asn17 on α1 of one monomer with Tyr93 on α3 and Tyr137 of α4 from the adjacent monomer (Fig. 2C and E).

Finally, an additional step involves transient contact with surface residues (for example, Gln11 and Asn220), after which the lipid diffuses into the bulk membrane.

### Retinal does not affect scrambling *in silico,* but polar residue substitutions along the cleft do

ActR scramblase activity is not changed in laboratory experiments with the addition of hydroxylamine, nor was illumination part of our protocol. Retinal was not present in our CGMD simulations. Thus, the scramblase activity of ActR does not depend on chromophore or light. Nonetheless, to assess whether retinal has an effect on scrambling rates *in silico*, we built a simplified bead model of retinal and included it in additional computational tests of scramblase activity (Fig. S2). The scrambling rate was not markedly different in these MD simulations from those without retinal.

In order to computationally evaluate the importance of the α1 and α4 polar amino acids as core elements of the scrambling groove, we created four ActR variants with two substitutions each and tested their scrambling activity as described above for the native sequence. Consistent with the identification of polar residues in the cleft as major contact points for the PO4 bead, both our double substitutions N17L/Y137F and N17L/T123L showed a reduced scrambling of ~1 event/µs (Fig. S2).

## DISCUSSION

Moonlighting is common among identified scramblases—multiple scrambling GPCRs (36, 44) and TMEM16 (45) in eukaryotes, numerous bacterial, ER, and mitochondrial membrane insertases (43, 46), and gram-positive (this work), gram-negative (32), and archaeal rhodopsins (20) perform other functions, including ion transport, protein translocation, and signal transduction. Members of the TMEM16 family of proteins, for example, function either as chloride channels or as nonspecific ion channels/lipid scramblases. The chloride channel members of this family may have derived from a primordial scramblase and evolved chloride transport at the expense of scramblase activity (47). It appears likely that yet-to-be-discovered scramblases moonlight with other functions. The ubiquitous nature of scramblase activity in diverse membrane proteins also helps explain why it has been difficult to pinpoint a unique and essential scramblase in some organisms (5).

Rhodopsins were ancient proteins found in early-Earth life forms (48, 49), suggesting they could have functioned as scramblases in the earliest cells. In the modern world, microbial rhodopsins are found across Bacteria, Archaea, and even some giant viruses (50, 51), with phylogenetic evidence suggesting that this wide distribution is due, at least in part, to frequent horizontal gene transfer (52). We are not aware of any conserved sequence motifs linked to the scramblase functionality of rhodopsins across the domains of life. This lack of sequence requirement suggests that scramblase functionality relies upon relatively hydrophilic surface characteristics without selecting for specific residues. Moreover, we do not expect opsin scramblase activity to be specific with respect to lipid head groups, as known scramblases are promiscuous for their substrates but do not have unlimited specificity for complex head groups (17, 53, 54). While scramblase activity is essential, the molecular surface underpinning this function appears to be under low selective pressure, possibly owing to the high rates of lipid scrambling exhibited by even rudimentary scramblases (55, 56) that may render scramblases easily evolvable.

Our simulations show that the ActR pentamer exhibits robust scrambling activity, consistent with our experiments showing that more ActR is needed to functionalize vesicles in comparison to bovine opsin. As monomeric and trimeric forms of ActR showed nominal scrambling, a fully functional, scrambling-competent hydrophilic groove is only present in the pentameric form. The dependence of activity on oligomerization aligns with observations from other scramblases such as VDAC1, where dimerization is necessary to form a translocation-competent groove (39), and BR, where trimerization generates interfaces that can scramble lipids (20).

The structural basis for actinorhodopsin-mediated scrambling centers on a hydrophilic groove formed by helices 1 and 3 + 4 of neighboring protomers. This pathway is lined with aromatic and polar residues that interact with the charged phospholipid headgroup to facilitate translocation. The orientation of the phospholipid during the scrambling event supports the credit-card insertion model, where the charged phosphate head group slides between helices, forming transient interactions with polar groups, and reorients in plane with the bulk lipid as it rejoins the bilayer (Fig. 2C, D, and F). Coarse-grained resolution limits the precision with which we can observe side chain orientation and hydrogen bonding. However, repeated observations of lipid contacts indicate a core set of residues—including Asn17, Phe21, Tyr32, and Tyr137—that play a role in the translocation mechanism.

We have shown that ActR from *Nanopelagicus ca*. of the acI lineage of actinobacteria is a phospholipid scramblase, making it the third known microbial opsin with scramblase activity alongside gram-negative bacterial PtqPR and archaeal BR. Our demonstration of the robust scrambling activity of gram-positive bacterial actinorhodopsins supports the notion that phospholipid scrambling is a fundamental function of rhodopsins across the domains of life. This insight provides a broad foundation for further study of the scramblase/opsin duality. Particularly intriguing open questions are (i) is there a pH dependence of scrambling activity? The oligomeric state of *Gloeobacter* rhodopsin has been shown to vary with pH (38), and our size exclusion chromatography mirrors these results (Fig. S2). Moreover, our computational results show that trimers do not scramble (Fig. 2B), thus providing a consistent model that the ActR low-pH oligomer is not an active scramblase. Decreased scramblase activity at low pH would prevent dissipation of the proton gradient; low phospholipid pKa values enable a scenario where outer leaflet phospholipids are protonated and subsequently deprotonated when scrambled to the inner leaflet (57). Though *Nanopelagicus ca*. primarily inhabits the freshwater epilimnion, a neutral pH habitat, freshwater lakes and bogs can contain low pH zones that may provide environmental pressure for a pH-dependent adaptation of scramblase activity by ActR (58). Perhaps this is a common mechanism for regulating bacterial rhodopsin scramblase activity with consequences for energetics as well as membrane biogenesis. (ii) Does the secondary antenna carotenoid associated with rhodopsin in many bacterial and archaeal opsins (23, 59–61) have an impact on scrambling? Further elucidation of the structural roles of secondary carotenoids as well as scrambling mechanisms is necessary to understand the interplay, if any, between antenna association and scramblase functionality. Although carotenoid association may add to thermal stability (60), our experimental and computational scrambling assays were all done in the absence of the actinorhodopsin carotenoid (23), and thus we can confidently say that the antenna is not required for scramblase activity. (iii) Is the earliest role of rhodopsin scrambling or photoheterotrophy, or did these activities co-evolve? Using our CGMD pipeline, we evaluated the scramblase activity of a plausible ancient opsin sequence recently proposed by ancestral sequence reconstruction (48). We did not observe scramblase activity (data not shown). While this model has very highly conserved residues associated with photoactivity, our current study concludes that scramblase activity relies more subtly on appropriate alignment of amino acids with relevant properties along a protomer interface than on invariant side chain identities. The study of the evolution of scramblase activity in early opsins is an exciting area that warrants additional analysis.

## MATERIALS AND METHODS

### Materials

Lipids including 16:0-18:1 PC (POPC) (1-palmitoyl-2-oleoyl-glycero-3-phosphocholine), 16:0-18:1 PG (POPG) [1-palmitoyl-2-oleoyl-sn-glycero-3-phospho-(1′-rac-glycerol) (sodium salt)], 16:0-06:0 NBD PC (C6-NBD-PC) (1-palmitoyl-2-{6-[(7-nitro-2-1,3-benzoxadiazol-4-yl)amino]hexanoyl}-sn-glycero-3-phosphocholine), and 14:0-06:0 NBD PC (1-myristoyl-2-{6-[(7-nitro-2-1,3-benzoxadiazol-4-yl)amino]hexanoyl}-sn-glycero-3-phosphocholine) were purchased as chloroform solutions from Avanti Polar Lipids. n-Dodecyl-β-D-maltoside (DDM) was purchased from VWR International. Bio-Beads SM2 Adsorbents were purchased from Bio-Rad. Amberlite XAD-2 and sodium dithionite were purchased from Sigma-Aldrich, and fatty acid-free BSA was from EMD Millipore. Gibson Assembly Master Mix was purchased from New England BioLabs. PhusionTM High-Fidelity DNA Polymerase Transcription reaction components, including S6 RNA polymerase, NTPs, RNase inhibitor, and buffer, were purchased from Promega Corporation. Translation reaction components, including WEPRO 8240H, Creatine Kinase, and SUB-Amix, were purchased from CellFree Sciences.

### Plasmid construction

DNA codon-optimized for expression in *E. coli* and encoding the amino acid sequence of ActR (ActR_L06_ from *Nanopelagicus ca*. single-cell amplified genome L06 [acI-B]) (23, 62) was cloned into a pEU-C-His flexiVector, chosen for its suitability for *in vitro* protein production, under the transcriptional control of the SP6 promoter (33). Insertion of the coding sequence for ActR extended with a C-terminal 6x-His tag into the pEU flexiVector was performed by amplifying plasmid fragments using PCR, followed by Gibson Assembly (63, 64). The same DNA sequence was also inserted behind the T7 promoter of a pET-28a vector using Gibson Assembly for expression in *E. coli*. Amplification of DNA fragments was performed using Phusion High-Fidelity DNA Polymerase. Gibson Assembly was performed using Gibson Assembly Master Mix. The molecular weight of the 6x-His-tagged ActR monomer is 29.7 kDa, with a predicted pI of 8.64.

### Large-scale cell-free ActR production

Large-scale transcription and translation reactions were carried out essentially as described by Aly et al. (33, 65) using low magnesium buffer (Cell Free Sciences) for transcription. Briefly, ActR-His_6_ protein was synthesized in a 12 mL 8240H wheat germ extract cell-free translation system (Cell Free Sciences) at 60 OD, using 0.1% maltose neopentyl glycol-10 with 0.02% cholesteryl hemisuccinate (CHS) buffer, and 0.1 mM all-trans retinal was added during the translation. ActR was purified by Ni-affinity chromatography followed by size exclusion. Protein was concentrated to approximately 15 mg/mL in 10 mM HEPES, pH 7.5, 100 mM NaCl, 0.3 mM TCEP, 0.05% DDM, and 0.01% CHS and stored at −80°C. The contaminating higher molecular weight bands were identified by mass spectrometry to contain uncharacterized wheat protein with Uniprot ID BAK08004.1, NADH Dehydrogenase, and HSP 90.

### ActR expression and purification in *E. coli*

BL21 (DE3) *E. coli* cells were used for recombinant expression of ActR. Ten milliliters of an overnight culture was used to inoculate 1 L of LB supplemented with 50 µg/mL kanamycin, which was then incubated with shaking (37°C and 250 rpm) until the OD_600nm_ reached 0.3, at which point the temperature was reduced to 22°C. At an OD_600nm_ of 0.5–0.6, ActR expression was induced by adding powdered IPTG to a final concentration of 0.5 mM. All-trans retinal was added concomitantly to a final concentration of 9 µM from a 50 mg/mL stock solution in ethanol. After 16–20 h of growth in the dark, the culture was centrifuged at 4,000 *g* for 12 min at 4°C. After a freeze-thaw cycle, cells were resuspended in lysis buffer (50 mM Tris, 300 mM NaCl, 5 mM MgCl_2_, 130 µM CaCl_2_, 1 mg/mL lysozyme, and 1 mM PMSF, pH 7.40) and sonicated. This and all subsequent steps were carried out at 4°C. The sample was clarified at 10,000 *g* for 20 min, after which the supernatant was centrifuged at 40,000 *g* for 45 min to pellet membranes. The membrane fraction was resuspended in 20 mL of solubilization buffer (50 mM Tris, 300 mM NaCl, 5 mM MgCl_2_, 130 µM CaCl_2_, and 10 mg/mL DDM, pH 7.40) using a Potter homogenizer and incubated with rocking in the dark for 16–20 h. Aggregates were removed by centrifugation at 40,000 *g* for 20 min. The supernatant (~20 mL), now containing solubilized membrane proteins, was collected and flowed continuously over 5 mL of Ni-NTA resin at 1 mL/min for 16–20 h using a peristaltic pump. The Ni-NTA resin was washed with >20 bed volumes of wash buffer (50 mM Tris, 300 mM NaCl, 50 mM Imidazole, and 0.5 mg/mL DDM, pH 7.40), and ActR was eluted with wash buffer containing successively higher imidazole concentrations, from 100 to 500 mM. ActR-containing fractions were pooled and concentrated along with DDM in a 10 kDa-cutoff spin concentrator (Millipore). Samples were dialyzed against 50 mM HEPES and 150 mM NaCl at pH 7.4 and injected onto a Superdex 200 Increase 10/300 GL size exclusion column pre-equilibrated with buffer identical to the dialysis reservoirs supplemented with 0.5 mg/mL DDM using an ÄKTA pure chromatography system. ActR-containing fractions were pooled, concentrated to approx. 0.5 mg/mL and immediately used for liposome reconstitution. The overall yield was ~0.5 mg/L of bacterial culture.

### ActR negative stain EM

ActR expressed in *E. coli* and purified through the metal affinity chromatography step was kept at 4°C and used to prepare EM grids on the same day. CF200-CU grids were glow discharged for 30 s at 15 mA on a Pelco easiGlow discharge system. Four microliters of sample (diluted in elution buffer to 240, 80, 32, or 8 µg/mL) was applied to each grid for 1 min before blotting. Grids were washed 2× in 20 µL buffer, 2× in 20 µL 2% uranyl acetate (UA), followed by 1-min incubation in 2% UA. All images were collected on a Talos L120c 120 keV electron microscope using a Ceta CMOS detector at the University of Wisconsin–Madison Cryo-EM Research Center.

### Reconstitution of ActR into liposomes

Liposomes were prepared using a 9:1 (mol/mol) POPC/POPG mixture, spiked with 0.5% 16:0-06:0 NBD PC or 14:0-06:0 NBD PC. The lipid mixture was prepared from chloroform stock solutions, dried under nitrogen or in a rotary evaporator, and then placed under vacuum overnight at ambient temperature before being resuspended in buffer (50 mM HEPES and 150 mM NaCl, pH 7.4) at a concentration of ~4 mg/mL. The resuspended lipids were processed further and taken for ActR reconstitution using either of two methods as follows. Method 1 (used for *E. coli*-produced ActR): the sample was bath sonicated for 5 min, incubated at ambient temperature for 1 h, sonicated again, and aliquoted for individual reconstitution experiments. Each aliquot was incubated with DDM (0.5% final) at ambient temperature for 1 h, rotating end-over-end. ActR was then added, and incubation was continued for 1 h. Reconstitution was achieved by adding washed Amberlite XAD-2 (150 mg), followed by incubation at ambient temperature for 1 h with end-over-end rotation. The samples were withdrawn and added to a fresh aliquot of Amberlite, followed by incubation overnight at 4°C with end-over-end rotation. Following this, the samples were withdrawn and transferred to tubes containing fresh Amberlite for a final incubation at ambient temperature for 1 h. The resulting proteoliposomes were stored at 4°C for up to 2 days, diluted 1/10 in excess buffer, and passed through a 0.2 µm filter on a Hamilton syringe assembly to produce a narrow size distribution of ActR-containing liposomes (66, 67). Method 2 (used for cell-free synthesized ActR): the sample was extruded successively through 400 nm and then 200 nm filters as described (39) to generate unilamellar vesicles with an average diameter of 170 nm. TX-100 (0.2% final) was added to destabilize the vesicles before adding ActR. The mixture was treated with one shot of washed BioBeads to remove detergent, initially at room temperature and then overnight at 4°C. The resulting vesicles were assayed directly (no further extrusion).

### Scramblase assays

Scramblase activity assays were performed as previously described (17, 20, 68, 69), using vesicles diluted to 2.5 mL in buffer (50 mM HEPES and 150 mM NaCl, pH 7.4). NBD fluorescence (*λ*_ex_ = 470 nm, *λ*_em_ = 530 nm) was monitored over time in spectrofluorimeters (QuantaMaster Model C-60/2000 or Horiba) equipped with a temperature-controlled cuvette holder, with a magnetic stirring apparatus. After obtaining a stable fluorescence signal, dithionite (from a 1 M stock) or fatty acid-free BSA (from a 75 mg/mL stock) was added. Addition of dithionite (final 10–20 mM) covalently modifies the fluorescent NBD moiety and eliminates its fluorescence signal (70), leading to fluorescence drops of 50%–100% for protein-free liposomes and scramblase-active proteoliposomes, respectively. BSA (final 15 mg/mL) captures and quenches NBD-PC molecules that desorb from the outer leaflet, leading to fluorescence drops of ~30%–60% for protein-free liposomes and scramblase-active proteoliposomes, respectively (8, 71).

Fluorescence traces were normalized to stable fluorescence emission values prior to dithionite or BSA addition and analyzed as described (72) using mono-exponential fits for protein-free liposomes and a double-exponential fit for ActR-containing samples. Analyses were performed within MATLAB or GraphPad Prism, using their curve-fitting functions.

### CGMD

An ActR monomer structural model was generated using Chai-1 (https://lab.chaidiscovery.com) for *de novo* structure prediction (73). The crystal structure of VDAC1 (PDB: 6G6U) and bovine opsin (PDB: 4A4M) was obtained from the Protein Data Bank (74–76). For scEMC3, missing regions in the crystal structure (PDB: 6WB9) were modeled using AlphaFold3 (https://alphafoldserver.com/welcome) (77, 78). Two BR dimers were tested and compared: one generated in AlphaFold3 and aligned to a crystal structure (PDB: 4MD2) and the other a vacuum-minimized crystal structure (PDB: 1AT9) (79, 80). We found similar scramblase activity in both models, and herein, we present the AI-predicted model. CGMD simulations were performed on a high-throughput computing cluster using the HTCondor software suite and GROMACS 2022.2 (https://manual.gromacs.org/2022.2) (81, 82). Visual Molecular Dynamics (https://www.ks.uiuc.edu/Research/vmd/) and Tachyon were used for trajectory visualization and image rendering (83, 84).

Protein models were initially subjected to energy minimization in a vacuum until the maximum force on any atom was below 100 kJ·mol⁻¹·nm⁻¹. Both the minimization and production runs were conducted using a GROMACS 2022.2 container image hosted on Docker Hub (https://hub.docker.com/r/gromacs/gromacs). Following minimization, the ActR trimer and pentamer were built by aligning the monomer to a trimeric *Halobacter salinarum* BR model (PDB: 1AT9) (80) and to a pentameric sodium-pumping rhodopsin (KR2) from *Dokdonia eikasta* (PDB: 6YC3), which was crystallographically determined at 2.1 Å resolution (37). VDAC1 monomers were aligned to the anti-parallel dimer PDB structure and subsequently manually adjusted to the highly scramblase-active parallel conformation described by Jahn et al. (39).

Proteins were embedded in a dummy membrane using the Orientation of Proteins in Membranes web server (https://opm.phar.umich.edu/ppm_server3) (85). The resulting protein–membrane systems were built and solvated using CHARMM-GUI (https://www.charmm-gui.org) and then converted to coarse-grained representations using the Martini 3 force field with Elastic Network Dynamics (86–90). Each membrane leaflet contained 400–500 molecules of 1,2-dioleoyl-sn-glycero-3-phosphocholine, for a total of 800–1,000 lipid molecules per bilayer. Electrostatic charges in the protein were counterbalanced by adding Na^+^ and Cl⁻ ions to the solution to bring the net charge to zero. Each system was solvated with approximately 2 × 10^4^ water beads.

To profile lipid dynamics in the presence of putative scramblases, simulations consisted of energy minimization, equilibration, and a production run with randomized initial velocities. Production simulations were run for 5 × 10^8^ steps with a time step of 20 fs, yielding a total trajectory length of 10 µs. Temperature was maintained at 310 K using a velocity-rescaling (v-rescale) thermostat within an NPT ensemble. Pressure was set to 1 bar using the Parrinello–Rahman barostat. Calculations were executed on a local GROMACS container and using HTCondor at the UW-Madison Center for High Throughput Computing (91).

Systems were interrogated for scramblase activity by measuring phosphate headgroup (PO_4_) angles of every DOPC every 1 ns, following the method described by Li et al. (43). A vector is defined whose direction bisects the angle between the two vectors from head group to lipid tail for each DOPC. A buffer region is defined from 55° to 125°, and an event is measured if the vector goes from under 55° to over 125° (or vice versa). Denoising was accomplished by averaging angles over 50 ns windows and sampling these smoothed data every 25 ns. The number of scrambling events per microsecond was averaged across all replicates. Simulations for VDAC1, scEMC3, BO, and ActR trimer were run twice, and all other models were run three times.

## SUPPLEMENTAL MATERIAL

Supplemental Material mbio.03278-25-s0001.pdf Figures S1 and S2.

ASM does not own the copyrights to Supplemental Material that may be linked to, or accessed through, an article. The authors have granted ASM a non-exclusive, world-wide license to publish the Supplemental Material files. Please contact the corresponding author directly for reuse.
