# Supplementary material for: Opsins are phospholipid scramblases in all domains of life
Source: mBio. 2025 Nov 26;17(1):e03278-25. doi: 10.1128/mbio.03278-25 (PMC12805836; doi:10.1128/mbio.03278-25)
Supplement: Supplemental Material — Figures S1 and S2. [file mbio.03278-25-s0001.pdf]

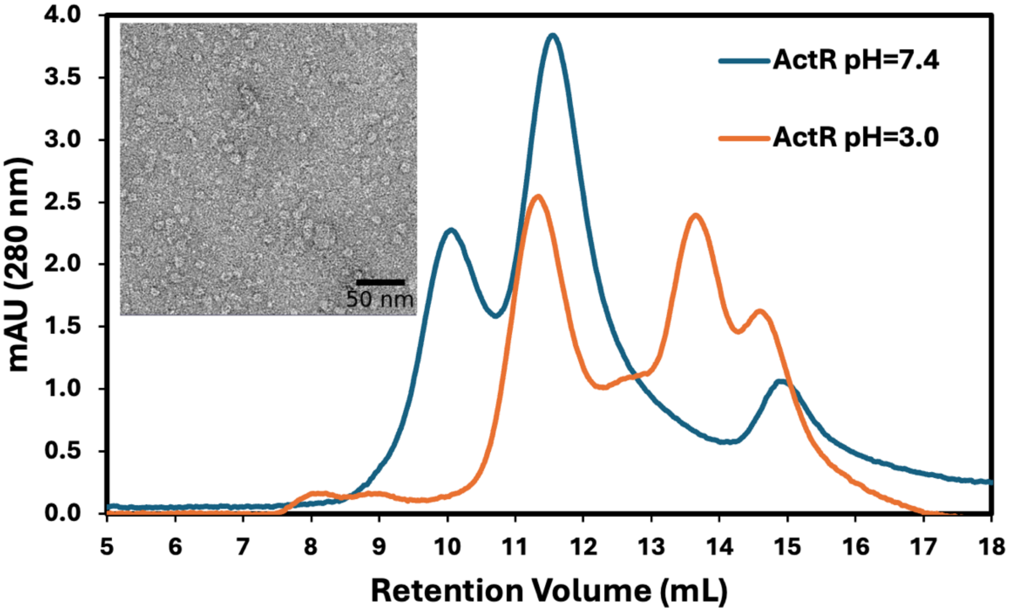

Supplemental Figure 1: ActR forms Pentamers at Neutral pH. ActR protein purified by nickel affinity was used for negative stain EM (inset shows grid area, isolated particles shown in Figure 2) and was run on an S200 Increase 10/300 GL SEC column at either pH 7.4 or 3.0. The peak at ~10 mL in the pH 7.4 trace is consistent with pentameric ActR. The oligomer equilibrium shifts to trimer and monomer at low pH, which is similar to behaviour reported for *Gloeobacter* rhodopsin (1).

ActR pentamer equilibrium is a function of pH

We investigated the oligomeric state of ActR and possible pH-dependent changes to this assembly using analytical size-exclusion chromatography (SEC). At pH=7.4, ActR predominantly assumes two oligomeric states as resolved by SEC (**Suppl Figure 2**). Based on expected retention volumes of soluble protein standards provided by Cytiva, these SEC peaks are consistent with pentameric and trimeric ActR. Furthermore, ActR changes the distribution of its oligomeric states at pH=3.0 (**Suppl Figure 2**), where the higher molecular weight, presumably pentameric, ActR peak disappears and ActR assumes a primarily trimeric state with a smaller molecular weight peak, possibly monomeric or dimeric ActR. Corollary experiments at pH=5.0 reveal similar SEC traces to those at pH=3.0 (not shown), consistent with the notion that the protonation state of a surface-exposed histidine residue such as H61 is responsible for controlling the stability of states. The mixture of oligomers at both pH=7.4 and pH=3.0 indicate that an equilibrium exists among oligomeric states of ActR. Stable oligomers ordinarily elute from a size-exclusion column in relatively narrow peaks. Contrariwise, ActR at all examined pH values exhibits relatively broad SEC elution peaks, suggesting that ActR oligomers exchange subunits over the course of size-exclusion.

To verify the pentameric state at neutral pH, electron microscopy images were taken of ActR following nickel affinity chromatography, and indicate a heterogeneous mixture of ActR oligomers (**Suppl Figure 2**). As the protonation state of H61 likely modulates the equilibrium of ActR oligomers with pH, the presence of imidazole in the sample probably effects the heterogeneity of conformations observed for ActR multimers. Nevertheless, ActR pentamers can be easily observed (**Figure 2**) and representative negative stain SEM images fit the profile of an AlphaFold2 model of pentameric ActR (**Figure 2**).

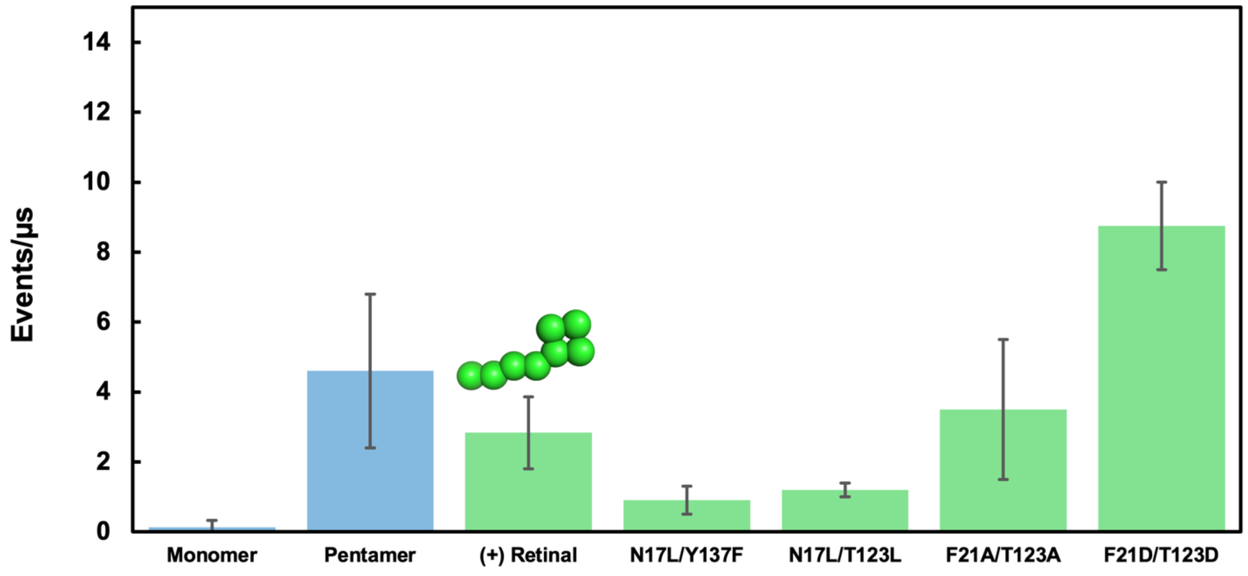

Supplemental Figure 2: CGMD Scramblase Activity Depends on Polar Residues in Protomer Interface. Monomer and pentamer rates are from Figure 2. The coarse-grained bead model for retinal is represented above the bar for scrambling rates with a retinal-bound opsin.

CGMD with retinal

A minimized all-atom pentamer model with retinal was used as the basis for positioning the CG ActR model, thus capturing the coordinates in order to subsequently create a GC retinal model. A python script was used to find centroids every 2-3 beads, 4 in the aliphatic tail and 4 for β-ionone ring of retinal. For bead definitions, SC (small) beads correspond to hydrocarbon backbones and TC (tiny) beads are typically used for rings because they allow for rigidity and tighter packing; the “1” is apolar, and the “2” is slightly more polar (maximum polarity is 4). The first bead of the tail is SC2, and the rest are SC1. All the ring beads are TC1. This approach created a simplified, space-filling model of the retinal ligand rather than a rigorous representation of a conjugated C20 ligand. During GROMACS production runs, a “pull-code” was used to keep the retinal ligands in place. This approach uses harmonic restraints between the centers of mass of two atoms to maintain a specified distance between them. Strong forces of 8000 kj/(mol*nm) were applied between bead 1 of the tail and the side chain bead of lysine 233, with a weaker force of 500 kj/(mol*nm)

between Gly136 and one side of the ring. Subsequent setup and MD runs and scramblase event counting were carried out as described in the main methods section. Result is the average of 3 x 2 μsec MD simulations.

CGMD with amino acid substitutions

The variant opsins were created by substituting side chains in PyMol, followed by vacuum minimization and subsequent steps as described in main text except that the membrane contained 800 DOPC molecules rather than 1000. All variants were tested in two independent runs with consistent outputs.

We designed three kinds of amino acid variants. First, in order to directly test the hypothesis that polar residues in the interface were important for interaction with the phospholipid head group we made two models that each had two amino acid substitutions in the scrambling cleft, from polar to non-polar side chains but of similar size. In one case the positions were at the top and bottom of the cleft on opposing subunits (N17L/T123L) and in the other both were at the top of the cleft, again on opposite faces (N17L/Y137F). Both of these reduced scrambling to <1 event/ μsec (**Suppl. Fig. 2**). As a check that not all substitutions would inhibit scrambling, two models were made with modest substitutions to alanine. In a double mutant (F21A/T123A) rates were not changed significantly. In a triple mutant (Y32A/Y93A/Y137A) scrambling was slightly higher; perhaps three substitutions compromise normal oligomer packing. Finally, introduction of two acidic side chains was tested (F21D/T123D), with an increase in scrambling rate. In our simplistic coarse-grained, implicit solvent model we neutralized each of these Asp residues by setting their charge to 0 in the parameter file and using the P2 polar bead type, reasoning that Asp side chains buried within the lipid bilayer have a higher pKa than those exposed to aqueous solvent. 10 Na+ beads were deleted to accommodate and balance the charge of the system. Future research might explore whether the higher apparent pKa of the carboxylic acid side chains within the membrane keeps these polar side chains protonated (uncharged) and, as a consequence, they may not repel the phosphate head groups but pull them through the cleft.

Reference

1. Morizumi T, Ou W-L, Van Eps N, Inoue K, Kandori H, Brown LS, Ernst OP. 2019. X-ray Crystallographic Structure and Oligomerization of Gloeobacter Rhodopsin. Sci Rep 9:11283.
